# Characterization of the complete chloroplast genome of *Tabernaemontana divaricate* (Apocynaceae), a valuable and endangered plant

**DOI:** 10.1080/23802359.2021.1984331

**Published:** 2021-11-03

**Authors:** Kun Zhang, Limin Liu, Xiaofei Shan

**Affiliations:** College of Life Sciences, Shanxi Datong University, Datong, China

**Keywords:** *Tabernaemontana divaricata*, Apocynaceae, chloroplast genome, illumina sequencing, phylogenetic analysis

## Abstract

*Tabernaemontana divaricate* is a vulnerable species of Apocynaceae with significant medicinal values. In this study, the complete chloroplast (cp) genome of *T. divaricate* was determined through the Illumina NovaSeq platform. The circular molecular genome was157,954 bp in length with two inverted repeats (IRa and IRb) regions of 25,769 bp, a large single-copy (LSC) region of 88,246 bp, and a small single-copy (SSC) region of 18,170 bp. It contained 131 genes, including 86 protein-coding, 37 tRNA, and eight rRNA genes. Phylogenetic analysis showed that *T. divaricata* exhibited the closest relationship with *Catharanthus roseus* and *Rauvolfia serpentina*.

The genus *Tabernaemontana* (Apocynaceae), comprising more than 100 species, is widespread in tropical and subtropical regions around the world (Li et al. 2019). *Tabernaemontana divaricate* (L.) R.Br. ex Roem. et Schult, also known as ‘Crape Jasmine,’ is an evergreen shrub and mainly distributed in southern China (Basavaraj et al. 2011). *T. divaricate* is an endangered species in the Redlist of China’s Biodiversity. As an important medicinal plant, *T. divaricate* is intensively used in antibacterial, antioxidant, analgesic, and antidiabetic treatments for its profusely high alkaloid content (Dantu et al. 2012; Anbukkarasi et al. 2016). In recent years, researches on *T. divaricate* have mainly focused on pharmacological properties (Naidoo et al. 2021), compounds identification (Li et al. 2019), and physiological mechanism (Thruppoyil and Ksiksi 2020). In this report, we characterized the complete chloroplast (cp) genome sequence of *T. divaricate* to contribute to further genetic and protective studies of this plant.

Fresh leaves of *T. divaricata* were collected from Chenshan Botanical Garden, Shanghai, China (31°08′N, 121°18′E). A voucher specimen (CSSH202105) was deposited in Shanxi Datong University (http://www.sxdtdx.edu.cn/, Kun Zhang, 876828320@qq.com). Genomic DNA was extracted according to the modified CTAB method (Doyle and Doyle 1987). After DNA purification, we constructed the libraries with an average length of 350 bp using the NexteraXT DNA Library Preparation Kit (Illumina, San Diego, CA, USA). High-throughput sequencing was performed on Illumina Novaseq 6000 platform, and the average length of the generated reads was 150 bp. Then 3.4 Gb clean reads were generated by editing raw sequence reads with NGS QC Tool kit (Patel and Jain 2012). The filtered reads were assembled by SPAdes v.3.11.0 software (Bankevich et al. 2012), and then the sequence of *T. divaricate* was annotated using PGA (Qu et al. 2019) with that of *Rauvolfia serpentina* (L.) Benth. ex Kurz (Accession number: NC_047244) as the initial reference. The annotated sequence has been submitted to NCBI (https://www.ncbi.nlm.nih.gov/nuccore/MZ073339.1/), the accession number is MZ073339.

The complete cp genome of *T. divaricate* was 157,954 bp in length and presented a quadripartite structure. It comprised two inverted repeats (IRa and IRb) regions, each of 25,769 bp, an 88,246 bp large single-copy (LSC) region, and an 18,170 bp small single-copy (SSC) region. The overall GC content detected in the *T. divaricata* cp genome was 38.1%. The assembled genome encoded 131 genes, including 86 protein-coding, 37 tRNA, and eight rRNA genes. Among the annotated genes, 15 genes (*atp*F, *ndh*A, *ndh*B, *pet*B, *pet*D, *rpl*2, *rpl*16, *rpo*C1, *rps*16, *trn*A-UGC, *trn*G-UCC, *trn*I-GAU, *trn*K-UUU, *trn*L-UAA, and *trn*V-UAC) contained one intron and two genes (*clp*P and *ycf*3) contained double introns. All genes occurred as a single copy, except that 10 genes (*ycf*1, *rrn*4.5, *rrn*5, *rrn*16, *rrn*23, tRNA-Ile, tRNA-Ala, tRNA-Arg, tRNA-Asn, and ORF302) were duplicated in IR regions.

To further reveal the phylogenetic position of *T. divaricata*, a phylogenetic analysis was conducted with nine complete cp genomes of Apocynaceae, 12 complete cp genomes within Asclepiadaceae family, and two species [*Hemerocallis citrina* Baroni, *Hemerocallis fulva* (L.) L.] from Asphodelaceae as outgroup. The sequences were downloaded from NCBI GenBank database and aligned using MAFFT (Katoh and Standley 2013). A maximum-likelihood phylogenetic tree was established by IQTREE v1.6 (Jana et al. 2016), which indicated that *T. divaricata* was sister to *Catharanthus roseus* (L.) G. Don and *Rauvolfia serpentina* (Figure 1). The information derived from this study provides a reference for future genetic and evolutionary surveys in *T. divaricata*, which may help facilitate the classification and conservation of this valuable and endangered plant.

**Figure 1.**
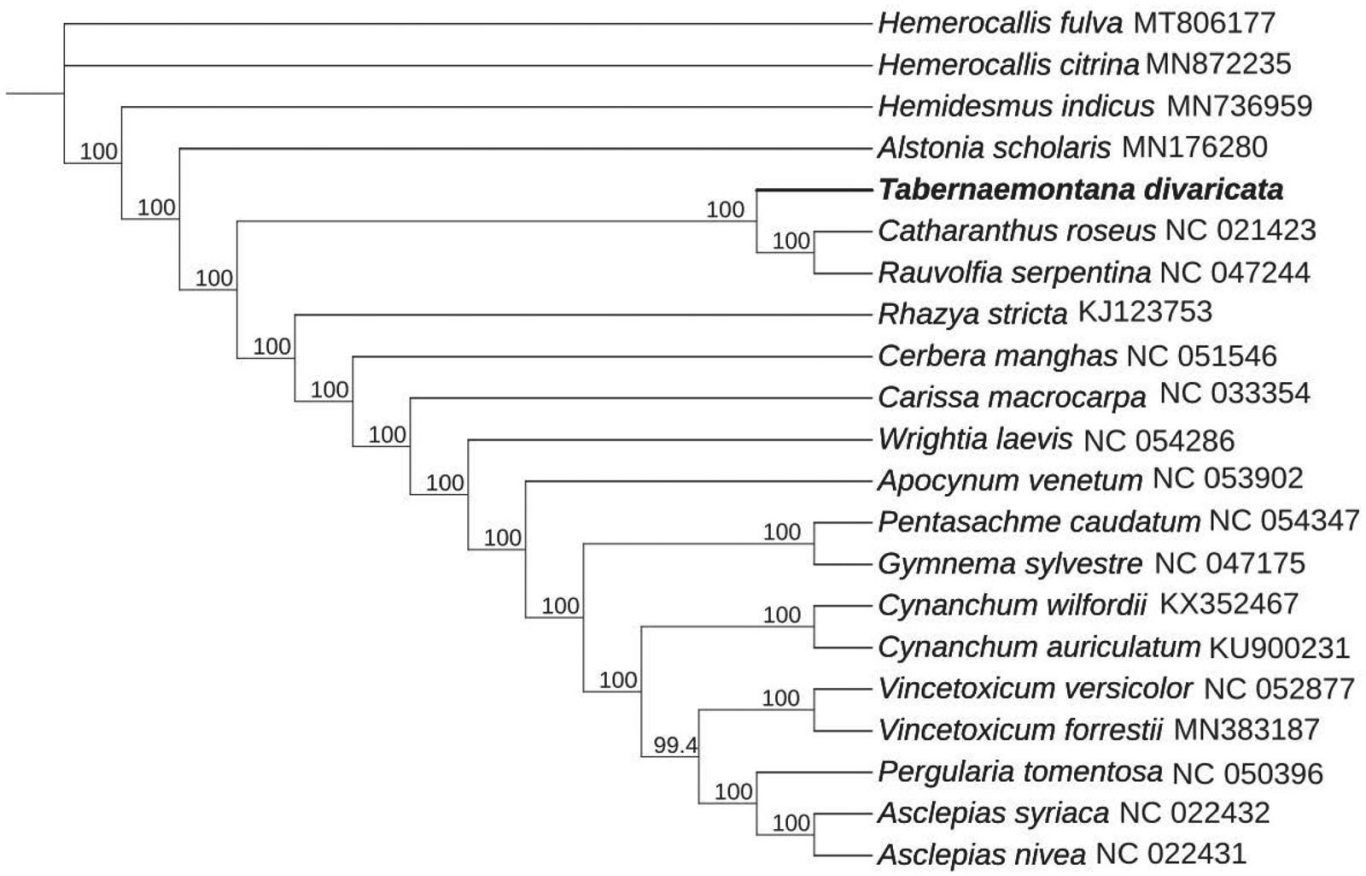
Maximum-likelihood phylogenetic tree for *T. divaricata* based on nine complete cp genomes of Apocynaceae, 12 complete cp genomes within Asclepiadaceae family, and two species (*Hemerocallis citrina* and *Hemerocallis fulva*) from Asphodelaceae as outgroup. The bootstrap values are located on each node and the Genbank accession numbers are shown beside the Latin name of the species.

## Data Availability

The assembled complete cp genome sequence of *T. divaricata* has been submitted to GenBank of NCBI and is openly available under the accession number: MZ073339 (https://www.ncbi.nlm.nih.gov/nuccore/MZ073339.1/). The associated BioProject, SRA, and Bio-Sample numbers are PRJNA726627, SRR14373633, and SAMN18951226, respectively.
